# A Role for Transportin in the Nuclear Import of Adenovirus Core Proteins and DNA

**DOI:** 10.1111/j.1600-0854.2007.00618.x

**Published:** 2007-07-30

**Authors:** Clemence E Hindley, Fiona J Lawrence, David A Matthews

**Affiliations:** Department of Cellular and Molecular Medicine, School of Medical Sciences, University of Bristol Bristol BS8 1TD, UK

**Keywords:** adenovirus, nuclear import, transportin

## Abstract

Adenoviruses target their double-stranded DNA genome and its associated core proteins to the interphase nucleus; this core structure then enters through the nuclear pore complex. We have used digitonin permeabilized cell import assays to study the cellular import factors involved in nuclear entry of virus DNA and the core proteins, protein V and protein VII. We show that inhibition of transportin results in aberrant localization of protein V and that transportin is necessary for protein V to accumulate in the nucleolus. Furthermore, inhibition of transportin results in inhibition of protein VII and DNA import, whereas disruption of the classical importin α–importin β import pathway has little effect. We show that mature protein VII has different import preferences from the precursor protein, preVII from which it is derived by proteolytic processing. While bacterially expressed glutathione S-transferase (GST)-preVII primarily utilizes the pathway mediated by importin α–importin β, bacterially expressed GST-VII favours the transportin pathway. This is significant because while preVII is important during viral replication and assembly only mature VII is available during viral DNA import to a newly infected cell. Our results implicate transportin as a key import receptor for the nuclear localization of adenovirus core.

Adenoviruses consist of an icosohedral particle containing a double-stranded DNA genome, which is condensed by association with adenovirus ‘core’ proteins called terminal protein, V, VII and Mu, all of which are contained within a capsid shell. Terminal protein is present as two copies; one attached to each of the 5’ ends of the linear genome of approximately 36 000 bp (1). Protein VII is formed by removal of 24 amino acids from the N-terminus of the precursor protein preVII (197 amino acids) by the viral protease following assembly of new virus particles. Mature protein VII is both the most abundant core protein and the most tightly associated with the viral DNA (2–4). Consequently, while preVII is involved in condensing the viral DNA during particle assembly, it is mature protein VII that is present in the viral DNA-core protein complex that enters the cell nucleus. Protein V (369 amino acids) is the next most abundant core protein and is thought to connect the core to the capsid (5,6). Finally, protein Mu (19 amino acids) has DNA condensing properties and is also associated with the viral DNA (4). Both protein V and protein VII have been shown to contain multiple nuclear import and nucleolar association signals (7–9).

Following entry into the host cell, the virion undergoes stepwise disassembly while trafficking through the cytoplasm, resulting in the partially degraded capsid docking at the nuclear membrane. Once docked the viral DNA, core proteins and some hexon (a component of the capsid) pass through the nuclear pore complex (NPC). A number of studies have attempted to identify soluble import factors that are likely to play a role in DNA import. Among the import receptors to have been implicated are the classical import factors known as importins α and β, which function as a heterodimer, and importin 7. Additional cellular factors have also been suggested to play a role including hsp70 and histone H1 (10,11).

The close association between the viral genome and the core proteins raises the possibility that the DNA is imported into the nucleus as a result of core protein import. However, to date no one has looked at the relationship between import factors, the major core proteins and the import of viral DNA. Therefore, we examined the interactions between the soluble import receptors and the major core proteins V and VII. Having identified import factors able to bind the core proteins V, preVII and VII, we used a permeabilized cell import assay and known competitors of the identified import receptors to confirm their import activity. This approach revealed striking differences in the import properties of preVII compared with mature VII. We also determined the effects of import receptor inhibitors on viral DNA import and correlated this with our observations of core protein import. We show that transportin is the primary import receptor for protein VII and the viral DNA and that transportin must be available for the correct subnuclear localization of protein V to occur.

## Results

### Core proteins are bound by more than one import receptor

**Figure 1:**
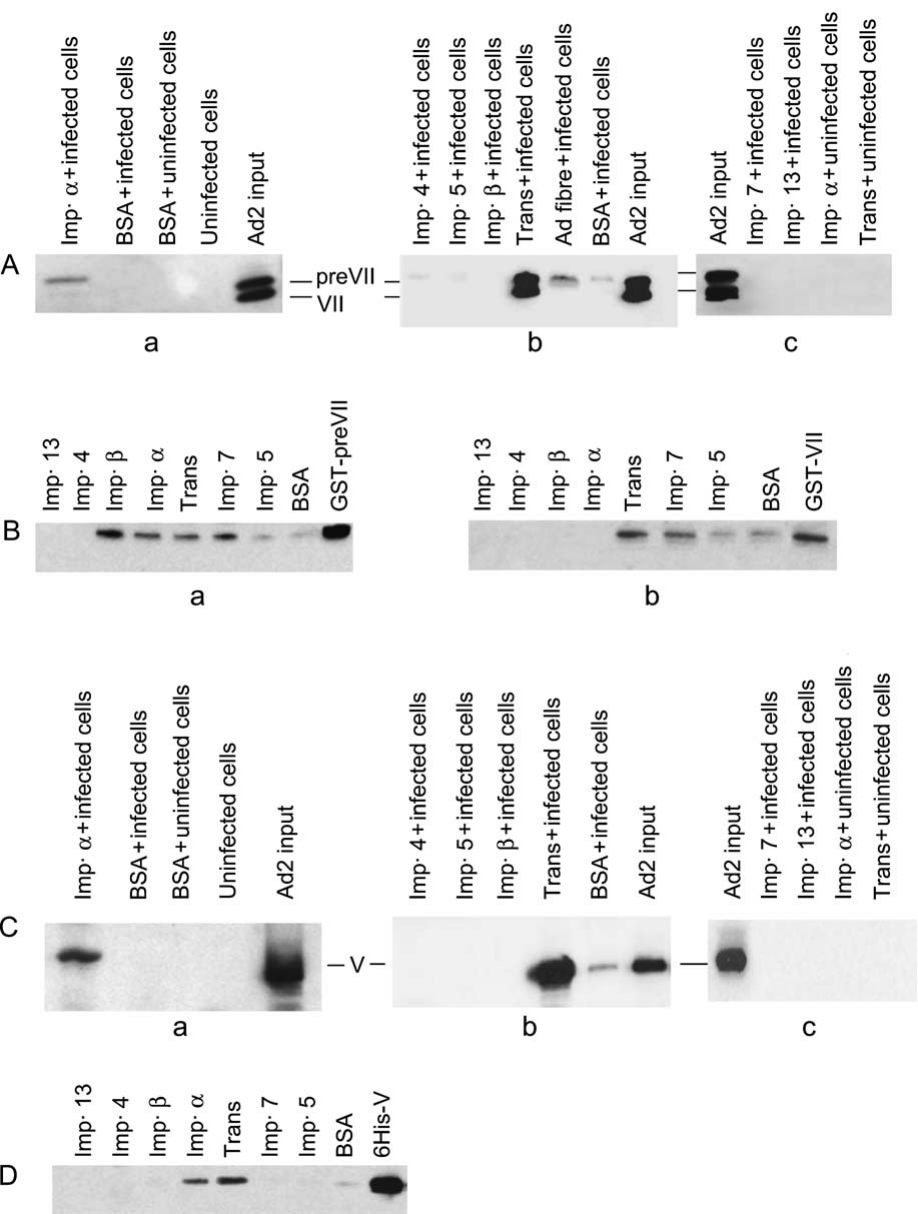
**Pull down of adenovirus core proteins by immobilized import receptors.** Purified recombinant import receptors or control proteins (BSA or purified recombinant adenovirus fibre protein) were immobilized on CN-Br-activated Sepharose beads. The immobilized proteins were then used to deplete cell extracts made from Ad2-infected HeLa cell cultures or from uninfected cell cultures as indicated (A and C). Alternatively, immobilized import factors were used to bind purified recombinant proteins GST-preVII (Ba), GST-VII (Bb) or 6His-V (D). Proteins bound to the immobilized import receptors were eluted and separated by SDS–PAGE. The adenovirus core proteins were detected by Western blotting using rat anti-VII (24) (A and B) and rabbit anti-V (6) (C and D). Secondary antibodies were horse radish peroxidase (HRP) conjugated and detection was by enhanced chemiluminescence. The position of preVII, mature VII and protein V is indicated. Imp., importin.

Purified recombinant import receptors were immobilized and used to deplete Ad2-infected and uninfected HeLa cell extracts. Following extensive washing, bound proteins were eluted and Western blotting performed using antibodies to the viral core proteins to identify interactions between import factors and viral proteins. Initially, we looked at the ‘classical’ pathway [which imports cargoes containing the simian virus 40 (SV40) T antigen nuclear localization signal (NLS)] by determining the ability of the adaptor protein, importin α, to bind the core proteins. We found that importin α was able to bind preVII but not VII (Figure 1Aa). We then investigated a much wider panel of import receptors and found that preVII and VII were bound by transportin (trans; which recognizes the M9 NLS) but not by importins β, 4 or 5 (Figure 1Ab). Finally, we determined that importins 7 and 13 also do not bind preVII or VII from infected cells (Figure 1Ac). We also used uninfected cell extracts as a pull-down control to ensure that the preparations of importin α and transportin did not fortuitously contain a protein that cross-reacted with the anti-VII antibody (Figure 1Ac).

We then examined the binding of bacterially expressed glutathione S-transferase (GST)-preVII and GST-VII to the immobilized import factors and found a wider range of binding. Thus, GST-preVII bound to transportin and importins α, β and 7 (Figure 1Ba), whereas GST-VII bound to importin 7 and transportin only (Figure 1Bb).

Similar analysis of protein V interactions showed that viral core protein V from infected cells bound to transportin and importin α only as did bacterially expressed 6His-V (Figure 1C,D, respectively). In light of these results, we concentrated on the transportin and classical import pathways because these import factors consistently bound core proteins in all the assays and, crucially, they were the only import factors which bound to viral core proteins derived from infected cell extracts.

### Establishing a permeablized cell import assay and the utility of competitive inhibitors to examine the classical and transportin-dependent import pathways

**Figure 2:**
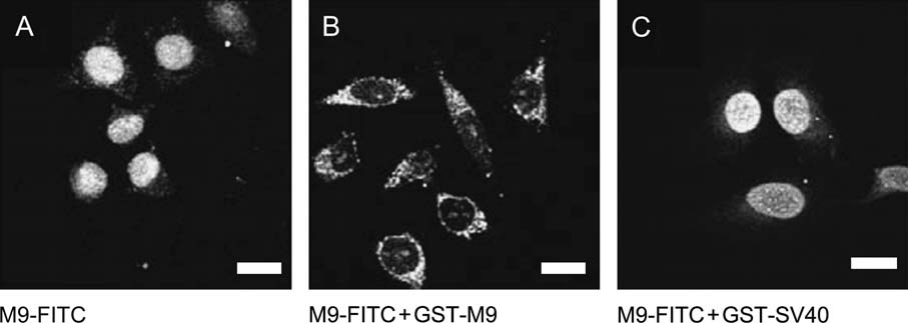
**Digitonin permeabilized cell import assay.** Complete import assays were carried out using HeLa cells permeabilized by incubation with digitonin. Cells were incubated at 30°C with complete reaction mixtures containing A) 500 nm FITC-M9. Then B) 2 mm GST-M9 or C) 2 mm GST-SV40 were added to the reaction mixtures as indicated. Following incubation, the localization of the FITC-M9 was determined by fluorescence microscopy. Bars represent 10 μm.

We used the digitonin permeabilized cell import assay (12) plus specific import receptor inhibitors to investigate the nuclear import of the adenovirus core. In order to confirm that the assay and nuclear import receptor inhibitors were functional in our hands, we first carried out import using fluorescein isothiocyanate (FITC)-labelled GST-M9 as a substrate. In a complete assay containing an energy-regenerating system and rabbit reticulocyte lysate (RRL) as a source of import receptors and other cellular proteins, FITC-GST-M9 was imported to the nucleus (Figure 2A). Addition of excess unlabelled GST-M9 inhibited this import (Figure 2B), whereas excess unlabelled GST-SV40 had no effect (Figure 2C). This confirmed that GST-M9 inhibits transportin-mediated import without affecting the classical import pathway. We also used FITC-GST-SV40 as import substrate and confirmed that addition of excess GST-SV40 inhibited its import, whereas addition of excess unlabelled GST-M9 did not (data not shown).

### PreVII and VII favour different import pathways for access to the nucleus

**Figure 3:**
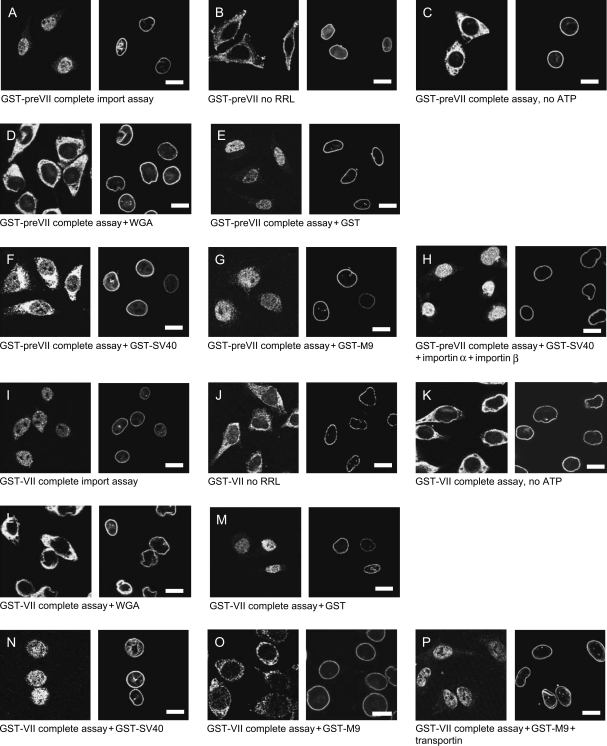
**GST-preVII and GST-VII import into permeabilized cells.** Complete import assays were carried out on HeLa cells permeabilized with digitonin. Cells were incubated at 30°C with complete reaction mixtures containing either (A–H) 500 nm GST-preVII or (I–P) GST-VII. For control reactions, either RRL or ATP was omitted from the reaction as indicated. Alternatively, 200 ng/mL WGA, 2 mm inhibitor (GST-SV40 or GST-M9), 2 mm GST, 1 mm importin α, 1 mm importin β or 1 mm transportin was added as indicated. Following incubation, the cells were fixed, permeabilized and stained for preVII and VII using rat anti-VII and costained for lamin A/C using mouse anti-lamin. Both were detected with alexafluor-conjugated secondary antibodies. In each pair of images, preVII/VII is shown on the left and lamin A/C on the right. Bars represent 10 μm.

Because preVII and mature VII (and their bacterially expressed counterparts) showed import-factor-binding preferences, we wanted to determine whether these discrepancies were reflected in an *in vitro* import assay. We found that purified GST-preVII was imported into the nucleus in a complete import assay (Figure 3A), but omission of RRL (Figure 3B), ATP (Figure 3C) or addition of wheat germ agglutinin (WGA) (Figure 3D) blocked import. Also, addition of GST alone to the import assay did not affect GST-preVII import (Figure 3E). When recombinant import inhibitors were added to the reaction mixtures, we found that GST-SV40 reduced nuclear import of GST-preVII (Figure 3F), whereas addition of GST-M9 had little effect upon nuclear import (Figure 3G). Addition of excess recombinant importins α and β to the GST-SV40-containing reaction restored import of GST-preVII (Figure 3H). Comparable examination of GST-VII import (Figure 3I–P) showed that, in contrast to GST-preVII, import of GST-VII still took place in the presence of GST-SV40 (Figure 3N), whereas GST-M9 had an inhibitory effect (Figure 3O). Addition of excess recombinant transportin to the GST-M9-containing reactions restored nuclear import of GST-VII (Figure 3P).

### Transportin is required for nucleolar localization of protein V

**Figure 4:**
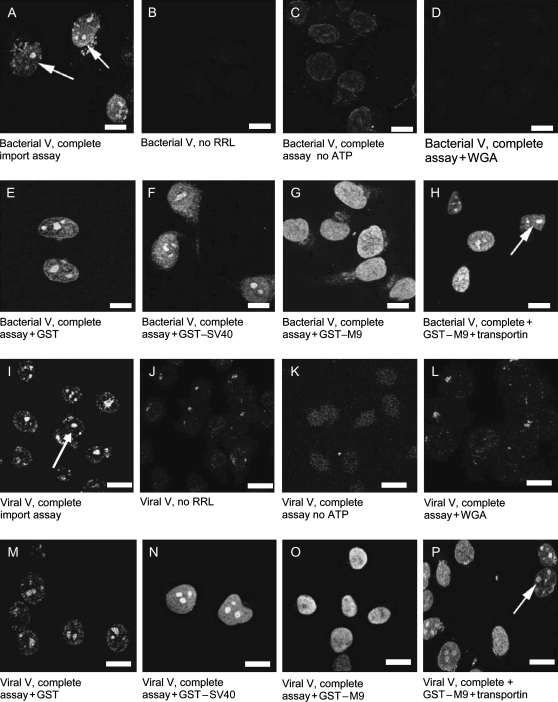
**V import into permeabilized cells.** Permeabilized HeLa cells were incubated at 30°C with complete import reaction mixtures containing either A–H) 500 nm bacterially expressed, 6His-tagged protein V or I–P) protein V derived from 2 × 10^8^ plaque-forming units heat-treated virus. For control reactions, either RRL or ATP was omitted from the reaction or 200 ng/mL WGA was added as indicated. To assess the contribution of different import factors to the import of protein V, either 2 mm inhibitor (GST-SV40 or GST-M9) or 1 mm transportin was added as indicated. Following incubation, cells were fixed, permeabilized and stained for protein V using rabbit anti-V (6) and alexafluor-conjugated secondary antibody. Arrowheads in these images point to nucleolar accumulation of protein V. Bars represent 10 μm.

We wanted to determine if protein V showed a preference for import factors and to examine if, as in adenovirus-infected cells (6), protein V localized to the nucleolus. In complete import assays, bacterially expressed, purified V was imported into the nucleus and showed strong nucleolar staining in approximately 50% of cells (Figure 4A). Omission of RRL (Figure 4B), ATP (Figure 4C) or addition of WGA (Figure 4D) reduced import of bacterial V. Addition of GST only (Figure 4E) or GST-SV40 (Figure 4F) had very little effect on import; V was still imported and was nucleolar in around half of cells. However, addition of GST-M9 consistently ablated nucleolar accumulation of V without inhibiting its nuclear import (Figure 4G). Moreover, including additional recombinant transportin alongside GST-M9 restored nucleolar accumulation (Figure 4H).

Import of V was also studied using purified adenovirus particles that had previously been incubated at 45°C for 10 minutes. The heat treatment causes partial disassembly of the capsid, mimicking the disassembly of the virus particle that occurs prior to its arrival at the NPC during a normal infection (13). Investigation of the import of protein V from these viruses confirmed the data obtained using purified bacterially expressed protein (Figure 4I–P). Finally, a mixture of GST-SV40 plus GST-M9 did not block import of viral or bacterial V to the nucleus, however, protein V did not accumulate in the nucleolus (data not shown).

**Figure 5:**
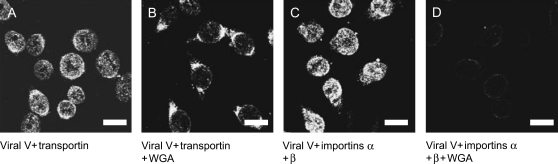
**Minimal import assay for protein V.** Cells were incubated at 30°C with minimal import reactions containing protein V from 2 × 10^8^ plaque-forming units heat-treated virus, 2 mm GTP and 2 mm Ran. Either 1 mm transportin or 1 mm each of both importin α and importin β were added as indicated. As a control, 200 ng/mL WGA was also added to duplicate experiments where indicated. Following incubation, the cells were fixed, permeabilized and stained for protein V using rabbit anti-V (6) and alexafluor-conjugated secondary antibody. Bars represent 10 μm.

We next used minimal import assays in which specific import receptors plus Ran, GTP and an energy-regenerating system were added to the permeabilized cells to further examine the import of viral protein V. The results demonstrated that protein V could be imported by transportin only (Figure 5A) or by importins α plus β (Figure 5C); however, V did not accumulate in the nucleolus in either case. WGA inhibited the import of protein V in both assays, confirming that the import observed was dependent on active transport through the NPC (Figure 5B,D).

### Virus DNA import is inhibited by GST-M9 and restored by transportin

**Figure 6:**
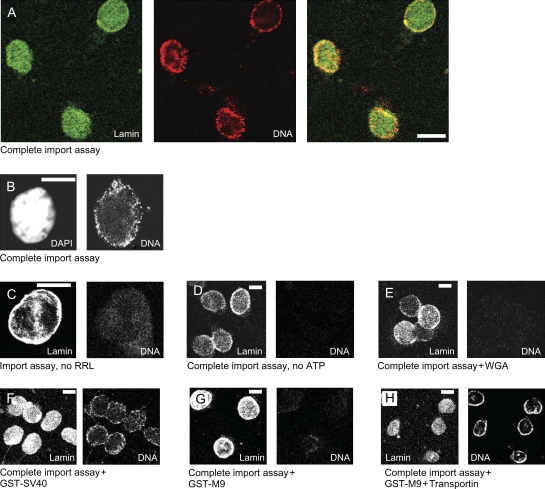
**Virus DNA import into permeabilized cells.** Permeabilized HeLa cells were incubated at 30°C with complete import mixtures containing 2 × 10^8^ plaque-forming units purified virus. In duplicate experiments, 2 mm inhibitor (GST-SV40 or GST-M9) and/or 1 mm transportin was added to the import mix as indicated. Following incubation, the cells were fixed, permeabilized and processed for detection of the viral DNA by FISH. Cells were costained for lamin A/C using mouse anti-lamin (Santa Cruz) and were mounted using vectashield mounting medium containing DAPI (Vector). A) A two-colour confocal laser micrograph image with lamin in green and viral DNA in red together with a merged image. In each subsequent pair of images, B) DAPI or C–H) lamin A/C staining is shown on the left and DNA is on the right. Bars represent 10 μm.

Import of the viral DNA genome was investigated using fluorescent *in situ* hybridization (FISH), which was carried out on permeabilized cells subjected to import assays using purified virus that had not been heat treated (we were unable to detect the DNA from heat-treated particles). Following the same approach and criteria as the initial studies of adenovirus DNA import (10), we took colocalization of viral DNA and lamin A/C, a component of the inner nuclear membrane, to indicate that import had taken place. In a complete import assay, DNA was seen to be concentrated in discrete foci overlapping the lamin staining (Figure 6A). As the lamin appeared to undergo some structural disintegration in cells that had undergone import, compared with negative controls, we also stained the nuclear DNA with 4′,6-diamidino-2-phenylindole (DAPI) to confirm that the viral DNA was in the nucleus (Figure 6B) and found that this was indeed the case. Viral DNA import was inhibited by omission of RRL (Figure 6C), ATP (Figure 6D) or by addition of WGA (Figure 6E) to the import reaction mixture. Addition of excess GST-SV40 to the import reaction mixture did not effect DNA import (Figure 6F), an observation that contradicts previous studies (10). In our assay, the transportin inhibitor GST-M9 prevented DNA import completely (Figure 6G) and addition of excess recombinant transportin to the import reaction overcame this block (Figure 6H). Following addition of purified V or GST-VII to the import reaction mixture, viral DNA was still seen in the nucleus (data not shown).

## Discussion

We have used digitonin permeabilized cells to investigate the nuclear import pathways utilized by the adenovirus genome and core proteins. In all cases, we found that import was dependent on the availability of soluble cellular components, energy and a functional NPC confirming that it is an active process.

A recent detailed examination by Wodrich et al. showed that recombinant preVII is able to interact with a range of import receptors, specifically importins α, β, 7 and transportin (9). Interestingly, although we see similar interactions with bacterially expressed preVII, we detect fewer interactions between import factors and preVII from infected cells; Wodrich et al. did not examine a mature VII construct. Our pull-down assay results indicate that the classical and/or transportin-mediated import pathways are likely candidates for the route of nuclear import of preVII and VII. To determine whether this was the case, we established an assay in which GST-SV40 and GST-M9 could be used as inhibitors of importin α and transportin, respectively.

We examined import of GST-preVII and GST-VII in complete assays using RRL as a source of proteins. Under these conditions, all the import pathways are available. We show that the nuclear import of GST-preVII is inhibited by the importin α inhibitor GST-SV40, whereas GST-VII import is inhibited by the transportin inhibitor GST-M9. These observations correlate to some extent with our pull-down data, which indicates that virally derived preVII, but not the mature VII, interacts with importin α and that mature VII only interacts with transportin. In addition, bacterially expressed GST-preVII interacts with importins α and β, whereas GST-VII does not.

The regions responsible for bacterially expressed preVII binding to individual import factors have been mapped and they are all present in mature VII (9). Despite this, our experiments show that GST-VII displays a clear preference for the transportin import pathway even when other import pathways are available (conversely, preVII prefers the importin α/β import pathway). Moreover, Wodrich et al. also noticed that GST-preVII only efficiently bound to transportin when importins α and β were unavailable (9), which adds weight to the idea that preVII has a bias for import factor binding. Such a preference towards one particular import pathway has recently been described for c-Fos. This protein can bind to both importin β and transportin in affinity assays, but in import assays, clearly shows a preference for transportin (14). Our data strengthen the argument for using competitive inhibitors, where available, to investigate the functional significance of interactions between import factors and their cargoes.

Our data also shed light on at least one effect of processing from preVII to VII after assembly of the virus particle. During adenovirus replication, preVII is expressed and imported into the nucleus where it associates with viral DNA and eventually helps to condense the viral DNA during assembly of new particles. Once assembly is complete, the viral protease cleaves preVII to produce VII. It is this mature form that is a component of the adenovirus DNA/core protein complex that is imported into the nucleus of the next host cell. We believe that preVII cleavage to VII alters the protein structure changing the way this protein interacts with and utilize import factors such as importin α/β and transportin. In support of this idea, a set of rules determining NLS recognition by transportin have been published that emphasize the importance of protein structure (15).

In our pull-down assays, viral protein V and recombinant bacterially expressed protein V interacted with both importin α and transportin, suggesting that, like preVII, it too is able to utilize a variety of import pathways. However, unlike preVII/VII, protein V is apparently able to utilize either import pathway equally and evidently does not have a preference during nuclear import. Moreover, failure of a combination of GST-SV40 plus GST-M9 to inhibit V import in complete assays implies that this protein associates with additional import factors/adaptors not examined in this work. Complete import assays resulted in the same nucleolar localization of V as seen in adenovirus-infected cells (6). Ablation of this nucleolar accumulation by GST-M9 implies that it is dependent upon the availability of transportin. In a minimal assay transportin alone does not result in nucleolar localization of V, therefore further soluble factors, probably transportin cargoes, must be required for V to accumulate in the nucleolus. This data implies that accumulation of V in the nucleolus is not a simple charge-based interaction with ribosomal RNA. Moreover, because protein V nuclear import is unaffected by GST-M9, which we have shown does block viral DNA import, we believe protein V does not play a direct role in viral DNA import. This conclusion is supported by the recent finding that human adenoviruses with the V gene deleted are compromized in their growth but still viable (16).

Investigation of the import of viral DNA indicates that it too is dependent upon transportin for entry into the nucleus. As for V, preVII and mature VII, import of DNA is dependent upon energy, cellular factors and a functional NPC. Addition of GST-M9, but not GST-SV40, to the import reaction mix completely abolishes virus DNA import: excess transportin overcomes this block indicating that it must be available for DNA to be imported. The fact that GST-VII was not able to block DNA import in complete assays indicates that viral cores outcompete bacterially expressed fusion proteins during import. Our data contrast with that of Saphire et al. (10) in which they find that import is blocked by excess BSA-SV40. We have no definitive explanation for this discrepancy but suspect that the reason may lie in a technical difference between our experimental protocols.

Taken together, our data showing that import of GST-VII is inhibited by competition with GST-M9; that mature protein VII from virally infected cells interacts with transportin and the fact that VII is intimately associated with the imported viral DNA (17) indicate that transportin is the primary import receptor required for virus DNA import.

We believe that the factors already identified as being involved in viral DNA import, importin 7, importins α and β, hsp70 and histone H1 (10,11) play a role upstream of viral DNA import. A model has been suggested whereby the virus particle docks at the NPC through interaction with CAN/Nup 214. Next, histone H1, importins α/β and importin 7 promote viral particle disassembly by interacting with the capsid protein hexon and importing a proportion of the hexon prior to DNA import (11). We would extend this model: after docking at the NPC, interactions between import factors/hsp70/histone H1 and viral components such as hexon promote the capsid disassembly/conformational changes required for protein VII and transportin to functionally associate and import viral DNA through the NPC. However, we were unable to reliably demonstrate that any combination of purified import factors could mediate import in a minimal import assay. Potentially, additional factors, besides a route across the NPC, are necessary for successful viral DNA import.

We have been able to correlate the role of transportin in DNA import with its ability to import GST-VII *in vitro* and to bind VII from infected cells. Viral protein VII has been strongly implicated in viral DNA import (9,17). Our data extend previous reports to propose that mature protein VII is the most likely mediator of DNA import and that this import is facilitated primarily by transportin.

## Materials and Methods

### Virus

CsCl gradient-purified recombinant Ad2 virus particles were from G. Sala-Newby, University of Bristol. This recombinant virus is deleted for E1 and E3 and is propagated in 293 cells.

### Cells and cell extracts

HeLa cells were maintained in DMEM supplemented with 10% foetal calf serum. Infected cell extracts were made following infection of cells at a multiplicity of infection of 10 and incubation for 48 h at 37°C. Cells were washed, then lysed in PBS, 1% (v/v) Nonidet P-40 by sonication. Lysates were cleared by centrifugation at 14 000 ***g*** for 30 minutes.

### Cloning

Glutathione S-transferase expression vectors were based on pGEX-4T-3 (GE Healthcare). The preVII and VII coding sequences were amplified from pFG140 (18) and cloned immediately downstream of the GST coding sequence. The plasmids encoding GST-SV40, GST-M9 and GST-IBB have been described previously (19,20). The His_6_-tagged expression constructs for V (6), wild type Ran (21), importin α(21), importin β(21), transportin (21), importin 4 (22) and importin 5 (23) have all been described previously.

### Protein expression and purification

Glutathione S-transferase fusion proteins were expressed in the *Escherichia coli* strain BL21 following the recommended protocol (GE Healthcare), except for GST-PreVII and GST-VII, which were induced with 0.5 mm isopropyl-β-D-thiogalactopyranoside (IPTG) for 3 h at 30°C, the cells resuspended in lysis buffer (540 mm NaCl, 2.7 mm KCl, 10.15 mm Na_2_HPO_4_, 1.75 mm KH_2_PO_4_, 10 mm MgCl_2_, 1% Triton-x-100: protease inhibitors), disrupted by sonication and cleared by centrifugation. Proteins were purified from the cleared lysates by incubation with glutathione–Sepharose beads (Sigma) for 3 h at 4°C, followed by elution with 10 mm glutathione. Purified proteins were dialysed against transport buffer (TB) (see *in vitro* transport assay) and stored at −80°C.

Ran and the His-tagged import receptors, α, β, transportin, 4 and 5, were purified as described previously. V-His_6_ was expressed in BL21 cells: cells were grown to an OD_600_ of approximately 0.6, in 2YT broth, at 37°C, an equal volume of ice-cold broth was added and the cells were induced with 0.5 mm IPTG for 3 h at room temperature. Cells were resuspended in lysis luffer (50 mm Tris–HCl pH 7.5, 500 mm NaCl, 10 mm MgOAc, 5 mm 2-mercaptoethanol, 5% glycerol, 0.5% Tween-20: protease inhibitors) and disrupted by sonication. Protein was purified from cleared lysate using Ni-NTA agarose (Qiagen) according to the manufacturer’s instructions. Purified V was dialysed against TB and stored at −80°C.

### FITC labelling

Purified GST-M9 and GST-SV40 were labelled with FITC using the EZ-Label FITC Protein Labelling Kit (Pierce) following manufacturer’s instructions.

### Pull-down assays and Western blotting

Purified import receptors were dialysed into coupling buffer (0.1 m NaHCO_3_, 0.5 m NaCl, pH 8.5) and attached to CN-Br-activated Sepharose (Sigma) following manufacturer’s instructions. Equivalent amounts of protein were used to deplete Ad2-infected HeLa cell extracts. Following extensive washing with PBS, bound proteins were eluted in SDS loading buffer and identified by Western blotting.

### In vitro nuclear import assay

HeLa cells were seeded on 10-well slides so that they reached approximately 70% confluence at the time of the assay. Cells were washed in PBS and permeabilized by incubation in 0.007% (v/v) digitonin in TB (20 mm Hepes-KOH pH 7.4, 110 mm potassium acetate, 4 mm magnesium acetate, 0.5 mm ethylenediaminetetraacetic acid, 1 mm DTT and phosphatidylinositol) for 5 minutes on ice. Cells were washed extensively in 30°C TB before application of 50 mL import mix to each well. Slides were then incubated in a humidified chamber at 30°C for either 45 minutes, for protein import, or 60 minutes, for DNA import. Import mixes consist of 20 mL RRL (Promega) dialysed against TB; an ATP-regenerating system (2 mm ATP, 1.6 mg/mL creatine phosphate, 5 U/mL creatine phosphate kinase); import cargo and TB to 50 mL total volume. For minimal import assays, cells were incubated with a reaction mix containing the ATP-regenerating system, 2 mm GTP, 2 mm Ran, import cargo, import receptors and TB to 50 mL total volume. Following incubation slides were processed either for immunofluorescence or for FISH as described below.

### Immunofluorescence

Following *in vitro* import cells were washed in TB, fixed in 4% (v/v) formaldehyde in TB at room temperature for 15 minutes and permeabilized in 1% (v/v) Triton-x-100 at room temperature for 5 minutes. Cells were blocked in 10% (v/v) foetal calf serum in PBS prior to incubation with primary antibody. Primary antibodies used were mouse anti-lamin A/C (Santa Cruz) at 1:200, rabbit anti-protein V at 1:400, rat anti-protein VII (a kind gift of H. Wodrich) at 1:300 and mouse anti-GST (Sigma) at 1:200. Fluorescently labelled secondary antibodies (Molecular Probes) were used at 1:400. Slides were mounted using Vectorshield mounting medium (Vector Laboratories). Fluorescent *in situ* hybridization was carried out as described previously (10). Imaging of cells was performed using a Leica confocal laser microscope and a 63× oil immersion lens at the University of Bristol MRC Cell Imaging Facility.
